# Pannexin channel and connexin hemichannel expression in vascular function and inflammation

**DOI:** 10.1186/s12860-016-0119-3

**Published:** 2017-01-17

**Authors:** Daniela Begandt, Miranda E Good, Alex S. Keller, Leon J. DeLalio, Carol Rowley, Brant E. Isakson, Xavier F. Figueroa

**Affiliations:** 1 Robert M Berne Cardiovascular Research Center, University of Virginia School of Medicine, Charlottesville, VA 22908 USA; 2 Department of Molecular Physiology and Biophysics, University of Virginia School of Medicine, Charlottesville, VA USA; 3 Departamento de Fisiología, Facultad de Ciencias Biológicas, Pontificia Universidad Católica de Chile, Santiago, Chile

**Keywords:** Connexins, Pannexins, Vasculature, Inflammation, Endothelium, Smooth muscle

## Abstract

Control of blood flow distribution and tissue homeostasis depend on the tight regulation of and coordination between the microvascular network and circulating blood cells. Channels formed by connexins or pannexins that connect the intra- and extracellular compartments allow the release of paracrine signals, such as ATP and prostaglandins, and thus play a central role in achieving fine regulation and coordination of vascular function. This review focuses on vascular connexin hemichannels and pannexin channels. We review their expression pattern within the arterial and venous system with a special emphasis on how post-translational modifications by phosphorylation and S-nitrosylation of these channels modulate their function and contribute to vascular homeostasis. Furthermore, we highlight the contribution of these channels in smooth muscle cells and endothelial cells in the regulation of vasomotor tone as well as how these channels in endothelial cells regulate inflammatory responses such as during ischemic and hypoxic conditions. In addition, this review will touch on recent evidence implicating a role for these proteins in regulating red blood cell and platelet function.

## Background

The vascular system is a complex network that, according to the structure and function of the vessels, can be divided into two compartments: the arterial and venous circulations, which are connected through the capillaries. Both compartments can be subdivided into different vascular segments. In the arterial circulation, conduit arteries, resistance arteries and arterioles can be recognized, and in the venous circulation, post-capillary venules, venules and veins can be distinguished [1–3]. All these vascular segments are designed to perform different, but complementary functions in order to provide oxygen and nutrients to all individual cells of the organism and dispose of metabolic wastes, which can only be achieved by fine regulation and coordination of vascular function along the different segments. As blood vessels are complex structures formed by several cell types, control of vascular function depends on timely and precise communication between the different cellular components of the vessel wall, mainly smooth muscle cells (SMCs) and endothelial cells (ECs) [4–6]. Nevertheless, blood vessels must also work in coordination with cells that are part of the blood, such as red blood cells (RBCs) and platelets [7, 8]. Thus, the control of vascular function depends on the fine communication between diverse cell types that are not always in direct contact each other.

One important mechanism of cell-to-cell communication is mediated by the release of autocrine/paracrine signals. Nitric oxide (NO), prostaglandins and ATP are widely recognized autocrine/paracrine signals that play diverse roles in the control of vascular function, such as regulation of vasomotor tone, smooth muscle proliferation, platelet aggregation, vascular permeability and leucocyte transmigration [9–12]. In addition, a signaling mechanism known as endothelium-derived hyperpolarization (EDH) has also been found to play an important role in the control of vasomotor tone [4, 9–14]. Although the biochemical identity of this signal is still controversial, it has been consistently found that the initiation of EDH signaling depends on the hyperpolarization of ECs by the activation of Ca^2+^-activated K^+^ channels of small (SK_Ca_) and intermediate (IK_Ca_) conductance, which, in the vascular wall, are only expressed in the endothelium [4, 9–13]. Additionally to autocrine/paracrine signaling, the direct cell-to-cell communication via connexin-formed gap junction channels make an important contribution to the coordination of function between the different cell types of the vessel wall and among distinct segments of the vascular network in the microcirculation [15–17]. It is interesting to note that ECs and SMCs are functionally connected by gap junctions, which led to hypothesize that the EDH signaling corresponds to direct transmission of the hyperpolarizing current generated in ECs to SMCs through a gap junction-mediated pathway [4, 13].

Remarkably, connexin (Cx) proteins do not form only gap junction channels, but also may form functional hemichannels (i.e. half of a gap junction channel) that connect the cytoplasm with the extracellular milieu. Communication of the intracellular compartment with the extracellular space through low resistance membrane channels formed by connexins (i.e. gap junctions) or the structurally related proteins termed pannexins (Panxs) has emerged as key pathways to command and regulate several paracrine signaling mechanisms involved in the control of vascular function [4, 18–21]. In this review, we discuss the structure, regulation and function of gap junctions, connexin hemichannels and pannexin channels in the vasculature and briefly in the circulating anuclear cells that regulate vascular function, red blood cells (RBCs) and platelets.

### Structure of Connexin and Pannexin Proteins

Connexin and pannexin proteins are thought to belong to the same superfamily as they share some physic-chemical properties, such as the charged nature of the extracellular loop and the polar residue distribution in the transmembrane helices [22]. However, it remains to be determined if they evolved from a common ancestor or if they evolved convergently. All connexins and pannexins possess a similar structure with 4 transmembrane domains, two extracellular loops, an intracellular loop, and both the amino and carboxyl termini located intracellularly [23]. Generally, either six connexins or six pannexins come together and form a channel, except for Panx2 that seems to form heptamers or octamers [24]. A connexin-based hexamer is referred to as a connexin hemichannel [25]. Two connexin hemichannels from adjacent cells are able to dock and form a gap junction channel. Pannexin oligomers form pannexin channels in the membrane, similar to connexin hemichannels; however, there is limited evidence in vertebrates for pannexin channels to dock with channels of neighboring cells to form functional pannexin-formed gap junction channels [26].

In the human genome, there are 21 known isoforms of connexins that were originally grouped into three subfamilies, α, β, and γ; however, recent analyses of the evolution of connexins have regrouped them into five subfamilies, adding δ and ζ [22]. Cx32, Cx37, Cx40, Cx43, Cx45 and Cx47 are expressed in the vascular system, where Cx37, Cx40 and Cx43 are alpha connexins, Cx45 and Cx47 are gamma connexins, and Cx32 is a beta connexin [22]. All connexin isoforms have conserved regions of the second transmembrane domain, within the amino termini, and conserved cysteine residues in the extracellular loops [22]. The cytoplasmic loop and carboxyl terminus are the least conserved regions between connexins allowing for differential regulation of each connexin isoform and thus participation in unique signaling cascades, especially when multiple isoforms are expressed within the same cell [22]. Unlike connexins, there are only three mammalian pannexin isoforms, Panx1, Panx2 and Panx3, which share conserved regions in their extracellular loops, intracellular loop, and amino and carboxyl termini [20, 22, 26]. Panx1 and Panx2 each have two splice variants while the connexins are suggested to have splicing variants of their 5’-untranslated region, which can impact the translation and function of the proteins [26, 27].

#### Connexin and pannexin expression in the vascular wall

Connexins and pannexins are expressed throughout the cardiovascular system. The expression levels within the vascular tree are dependent upon vessel type, localization within the vessel, and the species being examined. The expression differences between macrovessels vs. microvessels and arteries vs. veins are continuing to be examined; however, the specificity of antibodies and detection of low expression levels remain limiting steps to overcome. The majority of the data of pannexin and connexin isoforms and their unique localizations along the vascular tree comes from studies using rodent models or human primary cells. Thus, species-dependent differences also add another level of complexity to our understanding of the localization and function of these isoforms within the vascular wall. Below is a brief description of the current knowledge regarding the expression of connexins and pannexins within the vascular wall (Table 1).

**Table 1:** Expression pattern of connexin and pannexin isoforms in vivo along the vascular tree. EC: endothelial cells; SMC: smooth muscle cells. ‘no’ indicates that a protein has yet to be identified in those size vessels, not that the protein is necessarily absent. Expression pattern varies between vascular beds

Protein	Large Arteries	Resistance Arteries and Arterioles	Capillaries	Post-Capillary Venules and Venules	Veins	References
Endothelial Cell Expression						
Cx32	only in isolated primary cell line	only in isolated primary cell line	no	no (low levels in primary human cell lines)	yes	[43]
Cx37	yes	yes	yes	downstream side of valved-veins and in some ECs of the wall of non-valved veins	downstream side of valved-veins and in some ECs of the wall of non-valved veins	[28–30, 33, 36–39]
Cx40	yes	yes	no	no	generally no	[28, 29, 32, 33, 36, 37, 39]
Cx43	predominately areas of non-laminar flow	yes	yes	upstream side of valved-veins and in some ECs of the wall of non-valved veins	upstream side of valved-veins and in some ECs of the wall of non-valved veins	[28–30, 32, 33, 35–39]
Cx45	yes	no	no	no	no	[41, 42]
Cx47	no	no	no	only subset of ECs that compose the valves	only subset of ECs that compose the valves	[38]
Panx1	yes	yes	yes	yes	no	[35, 44–46]
Panx2	yes	yes	no	no	no	[44, 46]
Panx3	no	<100 μm diameter	no	no	no	[44, 45]
Smooth Muscle Cell Expression						
Cx32	no	no		no	no	[43]
Cx37	yes	yes		no	yes	[30, 33, 36, 37]
Cx40	no	generally no		no	no	[32]
Cx43	yes	yes		no	yes	[28, 30, 32, 33, 35–37]
Cx45	yes	yes		yes	no	[28, 33, 37, 42]
Cx47	no	no		no	no	[38]
Panx1	no	yes		yes	no	[44–46]
Panx2	yes	yes		no	no	[44, 46]
Panx3	no	<100 μm diameter		no	no	[44, 45]

### Connexin expression in the vascular wall

Cx43 is, by far, the most studied connexin. It has been well characterized and found to be expressed in the vascular SMCs of large conduit vessels, such as the aorta, in numerous species [28, 29]. The SMCs from mouse and human resistance arteries and veins can also express Cx43 [30–33]. Isolated primary human SMCs and ECs from arteries and veins express Cx43 [33–35]. In vivo, Cx43 is expressed in arterial ECs of larger arteries only in areas of non-laminar flow, such as branching points, as well as in arterioles and capillaries, such as in the brain [29–31, 36, 37]. In the murine vasculature, Cx43 is expressed by ECs of non-valved veins, such as the inferior vena cava, but in valved veins, such as the saphenous vein, is expressed solely in the ECs composing valves [36, 38, 39]. Together the literature indicates that Cx43 is expressed in both SMCs and ECs of both arterial and venous vessels; however, localization and expression levels are species- and vessel-specific. Cx43 is regularly shown to be expressed in cell lines; however, this expression may be increased or induced after the cells have been cultured because they are no longer under normal flow conditions or polarized with the physiologically appropriate neighboring cells.

Cx37 and Cx40 are also found broadly across the vascular network. Cx40 is predominantly expressed in ECs; however, a weak expression of Cx40 may also be observed in SMCs such as rat brain vessels [32]. Cx40 is rarely observed in the venous vasculature, although a few studies have found expression in murine umbilical cord vein, portal vein, and in human umbilical vein ECs (HUVECs) [33, 36, 39, 40]. Cx37 expression in SMCs has been demonstrated in arteries of various diameters from various organs as well as in isolated primary human SMCs [30, 31, 33, 36, 37]. It was specifically noted that Cx37 is not found in veins from the coronary or splenic circulations, although weak expression was found in veins from other sections of the vascular tree [36, 38, 39]. However, Cx37 is strongly expressed in ECs that compose the valves in valved veins such as the saphenous vein, but similar to Cx43, it is not expressed in the surrounding non-valvular ECs of valved veins [38]. Cx40 and Cx37 are both expressed in ECs of mice and rats throughout the arterial tree as well as in isolated human ECs [28, 30–34, 36, 37, 40].

Less information is available about the roles and localization of Cx32, Cx45 and Cx47. A weak signal for Cx45 has been observed in ECs from the bovine aorta [41], but Cx45 expression is predominantly in SMCs as well as in cultured HUVSMCs [28, 33, 37, 42]. Although expression of Cx32 in vivo has only been reported in ECs of large murine veins, the analysis of primary cultures of human ECs suggest that Cx32 has significantly higher expression in ECs from larger vessels, such as HUVECs and HAECs, as compared to ECs from microvessels, such as cultured human microvascular ECs (HMVECs) [43]. Cx47 has recently been found within a small subset of ECs that compose the valves in veins [38]. As the information about these connexins (Cx32, Cx45 and Cx47) is just emerging, further studies are necessary to determine their expression and functional roles within the vasculature.

### Pannexin expression in the vascular wall

Pannexin channels are expressed throughout the body. The expression of these channels continues to be explored, but recent data indicate that Panx1 is almost ubiquitously expressed in murine ECs within arteries, arterioles, and venules, as well as in isolated human cells including an immortalized brain EC line, human saphenous vein ECs (HSaVECs), and HUVECs [35, 44, 45]. Panx1 expression in murine SMCs occurs only in arterioles and venules, while Panx3 is only found in arterioles <100 μm in diameter [44, 45]. Panx2 has been observed in SMCs of the pulmonary artery of mice and in the SMCs of the rat middle cerebral artery (MCA) [44, 46]. Although mRNA of all three pannexin isoforms were found in the rat MCA, only Panx1 and Panx2 proteins were found in ECs of these vessels [46]. Lastly, as with any membrane bound protein that is expressed endogenously in low levels, it is rather difficult to detect Pannexin mRNA from small vessels. False-negative detection is a rather common occurrence and thus can not be taken to mean a lack of expression. For this reason, protein detection in small vessels in particular, is the most definitive way to detect Pannexin 1; this in conjunction with a knockout animal to validate expression. Overall, mouse and human expression appear to be the most consistent and reliable expression patterns, and thus physiologically relevant set of models, whereas the rat appears to be more random in expression.

#### Regulation of connexin and pannexin function in the vascular wall

Regulation of connexin hemichannels and pannexin channels can occur via changes in extracellular and intracellular Ca^2+^, pH or cell membrane voltage or via post-translational modifications (PTMs). PTMs are covalent protein modifications that alter protein function via the addition or removal of chemical functional groups to amino acid residues on the protein. A diverse array of PTMs exists, including phosphorylation, S-nitrosylation, glycosylation and ubiquitination, among others. These PTMs have individual effects on protein function but can also act in concert with other PTMs to regulate complex physiological responses [35]. Protein modifications by phosphorylation and S-nitrosylation are central regulators of vascular function due to the large influence of nitric oxide synthase and kinase-mediated signaling pathways during vasomotor responses in both arteries and veins [47, 48]. Research on connexin hemichannels and pannexin channels reveals evidence that the activity of these channels can be regulated by phosphorylation. Understanding how phosphorylation and S-nitrosylation modulates channel function is critical for understanding how connexin hemichannels and pannexin channels contribute to vascular homeostasis [49, 50].

### Phosphorylation of connexin hemichannels

In the blood vessel wall, connexin hemichannels and gap junction channels assist in coordinating vasoconstrictor and vasodilator signaling between smooth muscle and endothelial cells [51]. Overall, a large body of research has been focused on the regulation of connexin gap junctions by phosphorylation, but far less information is available on the expression and functional control of connexin hemichannels in the vasculature [52]. Recently, a number of reports have observed that phosphorylation events on connexin hemichannels can alter channel permeability in cell culture [53]. However, there is limited information about the functional regulation of connexin hemichannels in vascular cells. Connexin hemichannels have been identified on ECs and SMCs [54–56]. In these cell types, connexin hemichannel activation is linked to the release of ATP, a key signaling molecule in the blood vessel wall [9]. Although the mechanistic gating of connexin hemichannels by phosphorylation is unclear in the vasculature, studies in other cell types has suggested that hemichannels can be directly regulated by kinases. Cx43 is a well-established phospho-substrate for mitogen-activated protein (MAP) kinases, as well as protein kinase C (PKC), which have been extensively reviewed elsewhere [52, 57–59]. Additionally, phosphorylation of Cx43 at amino acid serine residue S368 has been shown to reduce hemichannel opening, decrease permeability, and change ion selectivity [60, 61]. This inhibitory effect was in part confirmed in the vascular endothelium, whereby hypoxia caused a reduction in ATP release, depletion of surface Cx43, and increased phosphorylation of Cx43 S368 [54]. It is interesting to note that the inhibitory effects of hypoxia on Cx43 hemichannels from cultured astrocytes have been linked to the dephosphorylation of Cx43 hemichannels by protein phosphatases, which suggests the existence of a complex PTM network in the regulation of Cx43 hemichannels by phosphorylation [52]. Nevertheless, future studies are needed to better understand how phosphorylation of connexin hemichannels modulates vascular function, especially in vivo.

### Phosphorylation of pannexin channels

**Fig. 1:**
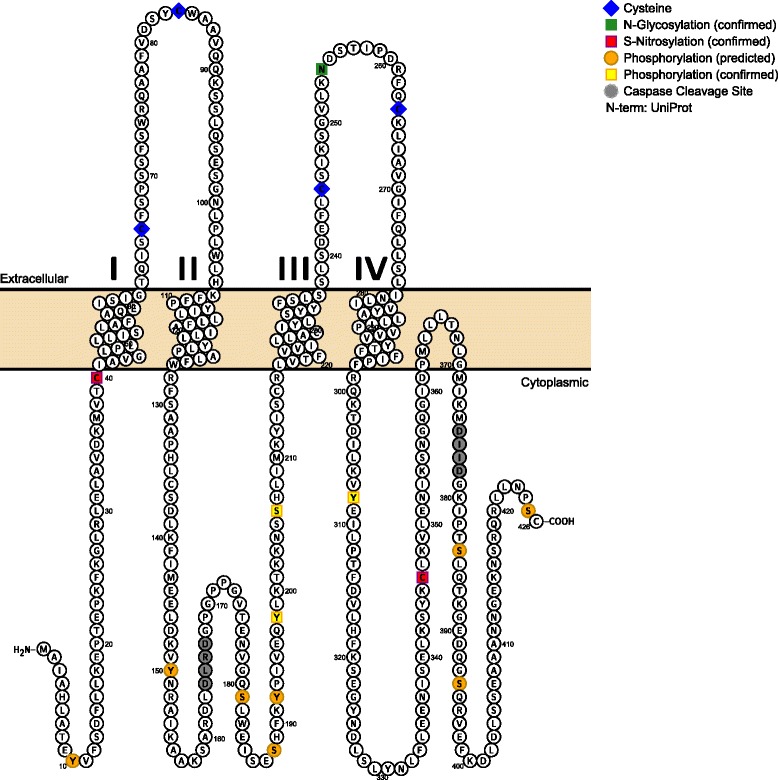
Diagram of mouse Pannexin 1 membrane topology and post-translational modifications. Based on sequence analysis using the UniProt database, Pannexin 1 is predicted to contain four transmembrane regions. It contains four conserved extracellular cysteine residues (*blue*) in the extracellular loops and a confirmed N-glycosylation site (*green*) necessary for plasma membrane translocation. Multiple post-translational modification sites by S-Nitrosylation (*pink*), phosphorylation (*yellow*), or proteolytic caspase cleavage (*gray*) have been confirmed to regulate channel gating. Additional post-translational modifications were predicted and annotated using PhosphoSitePlus (*orange*)

In addition to the presence of connexins in the vascular wall, pannexin channels have emerged as the most dominant regulator of extracellular purinergic signaling in vascular function [62–64]. A number of serine, threonine (T) and tyrosine (Y) phosphorylation sites have been predicted for pannexins based on their amino acid sequences, as well as putative recognition sites for protein kinase C (PKC), protein kinase A (PKA), and Ca^2+^/calmodulin-dependent protein kinase (CamKII) (Fig. 1) [65–67]. However, there is still a lack of direct biochemical evidence to link specific amino acid/kinase pairs with channel function. The current landscape of Panx1 PTM by phosphorylation is dominated by the regulatory role of tyrosine phosphorylation, which plays a unique and crucial role in regulating vascular function [68–70].

Mechanistically, a link between pannexin channel gating and tyrosine kinase phosphorylation was established using Panx1-expressing J778 macrophages and targeting the C-terminal Y308 amino acid of Panx1 in rodent hippocampal brain slices [71, 72]. In the vasculature, Lohman et al. recently confirmed the role for Src family kinase (SFK)-dependent tyrosine phosphorylation at residue Y198 of Panx1 in response to TNFα-receptor stimulation in the venous endothelium [45]. In this study, stimulation of ECs with TNFα resulted in an SFK-dependent increase in phosphorylation of Panx1 at Y198, which was paralleled by an increase in SFK activity [45].

Moreover, the Panx1 Y198 site was suggested to regulate SMC contraction and vascular tone in resistance arteries [62]. Based on earlier work that characterized a novel interaction between the α1-adrenergic receptor and Panx1-mediated ATP release, Billaud et al. demonstrated that pharmacological and genetic inhibition of the Panx1 intracellular loop motif containing Y198 prevents the Panx1 channel activation, ATP release, and vasoconstriction initiated by α1-adrenoceptor stimulation [62, 63]. In addition, this study showed that SMC-specific Panx1 deletion in mice, which exhibit blunted phenylephrine (PE)-stimulated vasoconstrictor responses, could be rescued by transfecting wild type Panx1 plasmids directly into arterial SMCs, but not by plasmids containing a mutated Y198 motif. These investigations suggest that Panx1 tyrosine phosphorylation within both ECs and SMCs could play a role in the regulation of vascular function [45, 62]. However, this has yet to be proven definitely and likely is a part of a much larger PTM set of events that have yet to be uncovered.

Outside of tyrosine phosphorylation, little is known about regulation of pannexin by serine/threonine PTM. In one study using pan-phosphoserine/threonine antibodies, the electrical stimulation of skeletal muscle was shown to enhance serine/threonine phosphorylation of Panx1 [73]. The increase in phosphorylation was associated with ATP release and dye uptake, which was sensitive to channel blocking agents. In a second study, the Panx1 residue S206 has been put forth as a putative serine phosphorylation site by protein kinase G (PKG) [74]. The inhibitory effect of the nitric oxide donor sodium nitroprusside (SNP) on Panx1 channel currents was shown to act through a cGMP-PKG dependent mechanism, and mutation of the serine at residue 206 to alanine blunted the SNP-dependent inhibition of Panx1 channel currents [74]. However, this investigation did not directly demonstrate substrate specificity of PKG for S206. In the future, it will be important to determine if S206 phosphorylation by PKG negatively affects Panx1 channel activity as Panx1-mediated ATP release has been shown to control α1-adrenoceptor-induced vasoconstriction and PKG signaling pathways are known to cause cessation of vascular SMC contraction [62, 75].

### S-Nitrosylation of connexin hemichannels

The addition of nitrosyl groups to cysteine (C) residues (S-nitrosylation) is another important PTM in both physiological conditions and disease [76, 77]. For connexins, there is clear evidence that NO can influence gap junction function in the vasculature, in both ECs and SMCs [78–81]. However, limited evidence exists for the regulated gating of connexin hemichannels by S-nitrosylation in the blood vessel wall. The mechanisms by which NO regulates connexin hemichannel activity have primarily been investigated in the central nervous system, mainly in astrocytes [53, 82]. However, it is important to note that both astrocytes and endothelial cells express the enzyme NO synthase (NOS), and thus, similarities may exist in the PTM mechanisms that regulate connexin hemichannel activity in these two cell types.

A number of studies have linked the regulation of connexin hemichannels with the production of NO. S-Nitrosylation has been proposed to regulate Cx43 hemichannels in astrocytes and cardiomyocytes, whereby conditions of oxygen deprivation and metabolic inhibition can enhance hemichannel activity as determined by pharmacology and in vitro [83, 84]. The potential modulation of connexin hemichannels by NO-mediated events has been linked to the functional regulation of vasodilator responses. Using connexin-deficient HeLa cells, Figueroa et al. demonstrated that Cx43, Cx40, and Cx37 could be stimulated to open when treated with NO donors and additionally could act as a conduit for NO transmission [78]. The study further demonstrated that acetylcholine (ACh)-induced vasodilation and the diffusion of NO from ECs to SMCs could be blocked in mesenteric arteries by inhibiting connexin-based channels. However, future studies are needed to determine the extent to which NO-activated connexin hemichannels contribute to the regulation of vascular function.

### S-Nitrosylation of pannexin channels

In addition to connexin hemichannels, evidence indicates that Panx1 channels can be regulated by S-nitrosylation. It was previously demonstrated that Panx1 channels can be activated by ischemic conditions in neurons, and that inhibition of the neuronal isoform of NOS (nNOS) during oxygen/glucose deprivation (OGD) blocks Panx1 channel activity in a NO-dependent and redox-sensitive manner [85, 86]. Direct evidence for a potential PTM site at Panx1 residue C28 was later identified in zebrafish using targeted mutagenesis experiments on cysteine residues in the intracellular and transmembrane domains of Panx1 [87]. Two later studies revealed additional residues (C40 and C346) as potential PTM sites of Panx1 [88]. Mutations of these two individual residues to serines resulted in a constitutively active channel [88]. Moreover, substitution of any of the four extracellular cysteine residues in Panx1 (C66, C84, C245, and C264) resulted in complete loss of channel function [89].

Although the previous studies did not identify Panx1 PTM by S-nitrosylation in a physiological context, they did set the stage for an investigation by Lohman et al, which demonstrated that multisite S-nitrosylation may be important for Panx1 channel gating in the vasculature [90]. The treatment of HEK and HAECs with the NO donor S-nitrosoglutathione (GSNO) induced Panx1 S-nitrosylation; however, instead of activating Panx1 as observed in neurons, NO had an inhibitory effect on channel currents and ATP release. This inhibition could be reversed by the reducing agent DTT. Furthermore, dual mutation of both C40 and C346 residues to alanine, but not either point mutation alone, was necessary to prevent GSNO-stimulated inhibition of Panx1 channel currents and ATP release. The results of this study highlight a potential negative regulatory mechanism of Panx1 channel gating by S-nitrosylation, which may balance vasoconstrictor responses produced by Panx1-mediated ATP release in SMCs of resistance arteries [62]. More recently, a similar investigation looking at the effects of NO donors on Panx1 activity in human embryonic kidney 293 (HEK) cells confirmed the observation of Lohman et al that NO donors closed Panx1 channels [74]. However, in this investigation, a PKG-dependent phosphorylation mechanism was suggested to participate in the control of channel activity, particularly by the stimulation of soluble guanylyl cyclase via NO. The mechanistic discrepancy between the two studies may be due to complex PTM regulatory signals, where multiple signaling cascades are involved in regulating pannexin channel gating as it relates to vascular function.

#### Connexin hemichannels and pannexin channels in the control of vasomotor tone

The regulation of blood flow distribution in the tissues relies on the well-integrated control of the microvascular resistance to blood flow by the adjustment of the diameter of resistance vessels, which depends on the level of constriction of SMCs (i.e. vasomotor tone). The contractile state of SMCs depends on the cytoplasmic Ca^2+^ concentration and Ca^2+^ sensitivity of the contractile machinery. Thereby, smooth muscle membrane potential plays a pivotal role in the regulation of vasomotor tone by temporally controlling the open probability of L-type, voltage-dependent Ca^2+^ channels [91, 92]. However, it is important to note that vasomotor tone is finally determined by the balance between a smooth muscle-dependent constrictor tone and an endothelium-dependent vasodilator tone [93, 94].

### Connexins and pannexins in smooth muscle cell function

The SMC-constrictor tone is mainly determined by the development of myogenic tone and the activity of the sympathetic system (i.e. sympathetic tone) [95, 96]. Myogenic tone is an intrinsic property of vascular smooth muscle and corresponds to the ability of vessels to develop a basal tone in response to the intravascular blood pressure [96, 97]. Interestingly, development of myogenic tone was found to be sensitive to blockers of connexin-based gap junctions in resistance arteries [98–100]. In small mesenteric resistance arteries, the myogenic response was blocked by 18α-glycyrrhetinic acid (18α-GA) and the connexin mimetic peptide ^37, 43^Gap27, which blocks channels formed by Cx37 or Cx43 [98]. Interestingly, the inhibition of the myogenic vasoconstriction was associated with a reduction in the pressure-induced SMC depolarization and the subsequent Ca^2+^ influx, which suggested that the inhibition was not related to synchronization of Ca^2+^ signaling, but rather to earlier signaling events such as the initiation of SMC-depolarization [98]. It is important to note that the effect of connexin mimetic peptides such as ^37, 43^Gap27 on connexin channels is time dependent. It has been suggested that application of these peptides for short periods of time (from 10 to 45 min) only inhibits hemichannel activity, without affecting gap junction communication, which are blocked only by much longer (>1 hr) periods of treatment [34, 101–103]. The myogenic response was evaluated only after 1 hr of treatment with ^37, 43^Gap27, thus the contribution of gap junction channels and hemichannels must be further explored. Connexin hemichannels are mechanosensitive and, therefore, may directly form part of the vascular smooth muscle pressure-sensitive mechanism involved in the development of myogenic vasoconstriction [104]. As Cx37, but not 43, was detected by immunofluorescence in SMCs of these mesenteric arteries, the activation of Cx37 channels was proposed to be involved in the pressure-induced smooth muscle cell depolarization that triggers the initiation of the myogenic response [98]. However, the resting basal vasomotor tone of cremaster muscle arterioles of Cx37 knockout (KO) mice was not different to that observed in wild type animals, thus the participation of other Cxs, like Cx43 and/or pannexins cannot be definitively ruled out [15].

In addition to the myogenic tone, connexin hemichannels may also involved in the sympathetic nerve-triggered vasoconstriction. The sympathetic vasomotor tone is primarily mediated by norepinephrine-evoked activation of α1-adrenoceptors in SMCs [95]. It was recently reported that blockade of connexin-based channels with 18β-glycyrrhetinic acid (18β-GA) or the Cx40 blocking peptide ^40^Gap27 attenuates the increase in perfusion pressure induced by PE in isolated, perfused rat kidney, an effect observed within a few minutes of peptide application [105]. Furthermore, the vasoconstrictor response was not altered by the connexin-blocking peptides ^37, 43^Gap27 or ^43^Gap26, a Cx43-specific channel blocker, suggesting participation of Cx40 hemichannels in the response [105]. The inhibition of the vasoconstriction observed in the presence of ^40^Gap27 suggests the participation of Cx40 hemichannels in the α1-adrenoceptor-mediated control of vasomotor tone; however, the mechanism involved has not been elucidated. This also highlights the serious problem of pharmacological reliance for determination of connexin hemichannel function. This therefor requires active investigation and the impetus is certainly on the connexin hemichannel field to definitely prove these exist in vivo and without the need to use vague inhibitors (e.g., as discussed by [106]).

The vasoconstrictor response initiated by α1-adrenoceptor activation in arterioles appears to be very complex and also involves autocrine signaling mediated by ATP release through pannexin channels. Recently Billaud et al. showed that stimulation of α1-adrenoceptors leads to Panx1 channel opening, which provides the pathway for ATP release [63]. The ATP released via Panx1 channels contributes, in great part, to the α1-adrenoceptor-mediated vasoconstriction through the activation of P2Y receptors [62, 63, 107]. Interestingly, this complementary vasoconstrictor mechanism is only coupled to α1-adrenoceptor activation while the response to serotonin or endothelin-1 is not affected by typical Panx1 channel blockers such as the Panx1 mimetic peptide ^10^Panx and probenecid, which was confirmed in the SMC Panx1-deficient mice [62]. This observation has now been confirmed independently pharmacologically [108] and genetically [109] in mouse mesenteric arterioles (as well as humans [data not shown]) highlighting the importance of the Panx1-α1-adrenerigc interaction, especially for translational outcomes.. The activation of Panx1 channels was found to rely on the specific amino acid sequence YLK, corresponding to residues 198 to 200 of the intracellular loop of Panx1 [45, 62, 63]. This suggests that phosphorylation at Tyr^198^ may mediate the α1-adrenoceptor-induced Panx1 channel opening, as recently reported by Lohman et al. to occur in the activation of Panx1 channels in venous ECs in response to TNFα [45, 62, 63].

### Connexins and pannexins in endothelial cell function

ECs play a central role in the regulation of vasomotor tone primarily by the production of Ca^2+^-dependent vasodilator signals such as NO, prostaglandins and EDH [4, 11, 12, 110]. Interestingly, NO and prostaglandins have been shown to be released through connexin hemichannels [78, 111]. It is important to note that NO may also induce the opening of hemichannels formed by the vascular connexins Cx37, Cx40 and Cx43, probably through S-nitrosylation, as demonstrated in the case of Cx43 hemichannels [78, 84]. In addition, several reports show that ECs may release ATP, a potent endothelium-dependent vasodilator, through Cx43 hemichannels and Panx1 channels [45, 54, 90, 112–117]. Therefore, endothelial ATP release may be involved in the control of vasomotor tone. Consistent with this hypothesis, endothelial Panx1-dependent ATP release was recently proposed to facilitate or enhance the EDH signal activated by ACh in ECs from mouse saphenous artery through a mechanism similar to that described for the response to PE in SMCs [118]. In this case; however, the increase of extracellular ATP concentration not only triggered the activation of P2 receptors directly by ATP, but also P1 receptors indirectly by the hydrolysis of ATP to adenosine, which was proposed to contribute to the ACh-induced increase in EC [Ca^2+^]_i_ [118]. Paradoxically, global deletion of Panx1 resulted in a selective reduction of the SK_Ca_ and IK_Ca_-initiated EDH-mediated vasodilation, without affecting the NO-dependent vasodilator component of the response to ACh, although both signals (EDH and NO) are Ca^2+^-dependent [118]. Ablation of Panx1 in these animals did not evoke a compensatory change in the expression of Panx2 or Panx3 in the saphenous artery; thus, the mechanism by which Panx1-driven ATP release activates selectively SK_Ca_ and IK_Ca_ in ECs will have to be addressed in further investigations [118].

#### Connexins and pannexins in the vessel wall inflammatory response

The role of connexin-based intercellular gap junction channels in inflammation has been investigated for decades. Multiple research groups have shown that connexin-based gap junctions play an important role regulating cell-to-cell communication during inflammation-related diseases such as atherosclerosis, hypertension or diabetes (for reviews see [4, 119, 120]). However, longstanding evidence also indicates that functional connexin hemichannels are involved in inflammation. While most research of connexin hemichannels in inflammation has been focused on the brain, by investigating astrocytes, neurons, and microglia (for reviews see [121, 122]), there is in vitro evidence for both pro- and anti-inflammatory roles for connexin hemichannels in the vasculature (Table 2). Pannexin channels that are highly expressed throughout the immune and cardiovascular system [45, 123, 124] are known to be key players during inflammation, but less is known about the mechanism by which pannexins participate in the cellular inflammatory response [125]. A role has been shown for pannexins in ischaemic conditions, pro-inflammatory neurotransmitter signaling, and surgically induced injuries on ECs that are presented below (Table 2).

**Table 2:** Pathophysiological conditions linked to connexin hemichannel or pannexin channel regulation. EC: endothelial cells; MS: mesangial cells; n.d.: not determined

Protein	Condition	Regulation	Cell type	Tissue origin	Species	Reference
Connexin hemichannels^a^						
Cx43	Gram-positive bacterial cell wall component peptidoglycan-induced immune response	Opening	EC	Brain	Mouse	[128]
Cx43	Hypoxia	Opening	EC	Brain	Rat	[124]
Cx43	Hypoxia	Closure	EC	Skin	Human	[54]
Cx43	Acute ischemic stroke mimicking (absence of extracellular Ca^2+^)	Opening	EC	Brain	Human	[35]
Cx43	Retinal ischemia-reperfusion injury	Opening	EC	Retina	Rat	[123]
Cx43	Retinal ischemia-reperfusion injury	Opening	EC	Retina	Rat	[124]
Cx43	Bradykinin-induced inflammation	Opening	EC	Brain	Rat/bovine	[34]
Cx43	Thrombin-induced inflammatory response	Closure	EC	Cornea	Bovine	[129]
Cx43	Diabetes (high glucose/cytokines treatment)-mimicking conditions	Opening	MS	Kidney	Mouse	[132]
Cx43	Surgical implantation of medical devices	Opening	EC	n.d.	Human/rat	[130]
Pannexin channels^a^						
Panx1	Acute ischemic stroke mimicking (absence of extracellular Ca^2+^)	Opening	EC	Brain	Human	[35]
Panx1	Cerebral ischemia-reperfusion injury	Opening	n.d.	Brain	Rat	[125]
Panx1	Pro-inflammatory neurotransmitter CGRP release	Opening	EC	Mesentery	Rat	[37]
Panx1	M-CSF-induced HSP-70 release immune response	Opening	EC	n.d.	Human	[126]
Panx1	TNFα-induced acute inflammation	Opening	EC	Diverse	Mouse/human	[45]
Panx1	Thrombin-induced inflammation	Opening	EC	Umbilical vein	Human	[112]
Panx1	Surgical harvesting of vessel	Opening	EC	Saphenous Vein	Human	[131]

^**a**^Note that the connexin hemichannel work has been proposed almost exclusively through pharmacological extrapolation, whereas the pannexin work combines genetic and pharmacological confirmation

### Role of connexin hemichannels and pannexin channels under inflammatory conditions

To date, there are only few studies carving out the distinct regulation between the activation of connexin hemichannel and/or pannexin channels. Kaneko et al. for example showed a role for both connexin hemichannels and pannexin channels in a blood–brain barrier (BBB) model system [35]. Using the human cerebral microvascular endothelial cell line hCMEC/D3, conditions mimicking acute ischemic stroke led to the opening of both Cx43 hemichannels and Panx1 channels, as observed by increased dye uptake and calcein efflux, respectively. Pharmacological inhibition with CBX or 18α-GA and knockdown with either Cx43 siRNA or Panx1 siRNA prevented the dye uptake and calcein efflux [35]. In another study, regulation of BBB ECs under inflammatory conditions was linked to connexin hemichannel opening; specifically bradykinin, a pro-inflammatory agent, evoked an increase in BBB permeability in rats through the activation of intracellular Ca^2+^ oscillations in ECs by a pathway sensitive to the Cx43 inhibitor Gap27, indicating the involvement of hemichannel opening and purinergic signaling in response to bradykinin [34].

Ischemic injuries resulting in inflammation also induce the opening of connexin hemichannels. In retinal ischemia-reperfusion (I/R) injury in rat, the opening of Cx43 hemichannels and gap junctional communication was shown to play a role in inflammation, vascular permeability and neuronal death [126]. In a similar study, I/R led to fragmented vessels, increased Evans Blue leakage into the retina, increased Cx43 expression, co-localization of activated astrocytes with ECs, and glial fibrillary acidic protein (GFAP) de-organization close to blood vessels resulting in retinal ganglion cell death [127]. These effects were prevented by treatment with Cx43 mimetic peptide during reperfusion, which strongly supports a Cx43 hemichannel-dependent mechanism during I/R injury [127]. Cerebral I/R injury in rats induced activation of astrocytes, microglia and further subsequent inflammatory responses. Treatment of the animals before or even after the induced ischemia with different doses of probenecid, a pannexin inhibitor, prevented I/R injury-induced inflammation and cell death by preventing increased expression of cathepsin B and calpain-1 and by increasing heat shock protein 70 (HSP70) release [128, 129]. Although these studies were done in different organs, namely retina and brain, they suggest a role of connexin hemichannels and pannexin channels in the I/R injury.

Hypoxia alone also appears to independently regulate connexin-based hemichannel opening in vitro. In rat brain microvascular endothelial R840K-05a cells, hypoxia-induced cell death could be rescued by the application of hemichannel inhibitors carbenoxolone (CBX), La^3+^ or Cx43 mimetic peptide [127]. Interestingly, astrocytes at penetrating vessels in the medulla oblongata release ATP via Cx26 hemichannel under hypercapnia (elevated arterial *P*_*CO2*_) to signal for breathing regulation [130]. Both conditions, low O_2_ levels and increased CO_2_ levels, signal via connexin hemichannel-mediated ATP release. However, studies also showed that in human microvascular ECs of dermal origin, induction of hypoxia for 48 h attenuated Cx43 hemichannel-mediated ATP release. In this study, Cx43 transcript, total, and surface protein levels were all reduced, and Cx43 showed higher phosphorylation at S368 (which leads to a closed state of this hemichannel) [54]. The regulation of Cx43 hemichannel activity as well as a potential role of pannexin channels in hypoxic conditions, remains to be investigated.

Exposure to pathogen-associated molecular patterns (PAMPs) causes the opening of Cx43 hemichannels in ECs of the BBB, which induces an early inflammatory process. Incubation of b.End5 cells, a murine brain endothelial cell line, with the gram-positive cell wall component peptidoglycan for 24 h induces an ATP release via PKC-dependent Cx43 hemichannel opening, resulting in increases in mRNA expression levels of Cx43, Interleukin-6 (IL-6) and Toll-like receptor 2 (TLR2). These results demonstrate the role of Cx43 hemichannels in early inflammatory response, which occurs before changing the inflammatory related gene expression [131]. Another pro-inflammatory TNFa-induced ATP release was shown to be Panx1-dependent. Lohman et al. recently identified an important mechanism of regulation of endothelial Panx1 channels in TNFα-induced inflammation ex vivo, using dissected mesenteric veins, and in vitro, using isolated human primary venous ECs [45]. Application of TNFα induced, within minutes, ATP release into the lumen of cannulated veins or into the media of cultured cells. Importantly, this effect was only seen in veins or venous ECs, but not in arteries or arterial ECs. This ATP release was Panx1 channel-mediated as shown by prevention of ATP release with pharmacological inhibitors, such as CBX or ^10^Panx, and with siRNA-mediated knockdown of Panx1. siRNA knockdown of Cx43, conversely, had no effect. Furthermore, cannulated veins from EC-specific Panx1-deficient mice failed to release ATP following TNFα stimulation. TNFα induced SFK-dependent phosphorylation at tyrosine Y198 in the intracellular loop of Panx1, leading to the opening of the channel. Intravital microscopy demonstrated that TNFα induced leukocyte adhesion and emigration was blunted in EC-specific Panx1-deficient mice [45]. In a similar study, thrombin induced a robust, Ca^2+^-dependent ATP release via Panx1 channels in HUVECs [115]. Pro-inflammatory mediators like thrombin or histamine were also reported to induce the inhibition of Cx43 hemichannel-mediated ATP release by either a rapid and transient RhoA activation or the activation of phospholipase C (PLC), depending on which G protein was expressed in the cell [132]. These results highlight Panx1 as well as Cx hemichannels as a key protein for the crosstalk between cytokine and purinergic signaling in early inflammation.

In addition to known vascular diseases, surgical implantation of medical devices can also trigger inflammatory responses that involve connexin hemichannel activity. This inflammatory response leads to thickening of the implant/muscle interface and increased numbers of infiltrating neutrophils [133]. Application of a Cx43 mimetic peptide (JM2), which binds to the microtubule binding domain of Cx43, resulted in the inhibition of Cx43 hemichannel-mediated ATP release in cultured HMVEC [133]. When applied to rats with a silicone implant, the JM2 peptide restored the matrix formation and cellularity surrounding the implants and reduced the number of infiltrated neutrophils, suggesting that blockade of Cx43 hemichannel opening and subsequent ATP release into the vasculature could reduce inflammation and improve tissue healing after implant surgery [133]. Similar to Cx43 hemichannel regulation during surgical intervention, surgical harvesting of human saphenous veins induced endothelial and/or smooth muscle injuries resulting in cellular dysfunction and intimal thickening, possibly due to ischemia in the vein following extraction. Intimal injuries during surgery led to ATP release, which evoked P2X_7_ receptor and/or Panx1 channel activation with subsequent P2X_7_ receptor downstream signaling. Pharmacological inhibition of Panx1 and P2X_7_R with probenecid and oxidized ATP, respectively, prevented inflammatory intimal hyperplasia and, therefore, could be a possible therapeutic target for improving successful grafting of vessels [134].

A functional role of Panx1 was also suggested in the signaling between the nervous and vascular system under pro-inflammatory conditions. Stimulation of primary arterial endothelial mesenteric cells and intact mesenteric arteries with the neurotransmitter calcitonin gene-related peptide (CGRP) leads to Panx1 channel opening as shown by ethidium uptake, which was prevented by the specific inhibition of CGRP receptors with the peptide CGRP_8-37_ or the blockade of Panx1 channels with probenecid. Furthermore, stimulation of perivascular sensory nerves with capsaicin led to CGRP release and downregulation of endothelial NOS (eNOS) expression, linking pro-inflammatory induced neurotransmitter release to the development of endothelial dysfunction during inflammation via decreased eNOS protein levels [37]. A possible role for connexin hemichannels was not investigated.

The development of renal and cellular alterations during diabetes in rats has been linked to increased Cx43 hemichannel opening in mesangial cells, specialized cells surrounding blood vessels in the kidney. Mouse MES-13 mesangial cells treated with high glucose and cytokines (tumor necrosis factor α (TNFα), IL-1β) for 48 h displayed an increase in cellular permeability that correlated with an enhanced uptake of ethidium bromide, suggesting an increase in connexin hemichannel opening [135]. However, pannexin channels cannot be ruled out and necessitate further pharmacological or genetic experiments. Taken together the data predominately suggest that preventing/reducing connexin hemichannel or pannexin channel opening may reduce inflammation in response to various stimuli.

#### Connexins and pannexins in anuclear circulating blood cells

##### Pannexins in RBCs

Long viewed simply as carriers of oxygen, in the past two decades red blood cells (RBCs) have received renewed attention for their potential ‘erythrocrine’ function; i.e., the ability to influence their own distribution across the vasculature by participating in signaling pathways that result in the modulation of vascular tone, thus controlling blood flow to tissues with relatively greater or lesser need of oxygenation [7, 136, 137]. Ellsworth et al. proposed in 1995 a pathway involving RBC release of ATP into the vessel lumen based on the knowledge that RBCs release ATP in response to hypoxic conditions and that ATP application results in significant increases in RBC supply rate to both arterioles and venules at the end of a capillary network [136, 138]. ATP released by RBCs is thought to bind to G protein-coupled P2Y receptors on the endothelium, initiating a signaling cascade to produce and release peripheral vasodilatory signaling molecules such as NO and prostacyclin [136]. Upstream transmission of this vasodilator response allows blood flow to be modulated to match local oxygen demand [139]. ATP is released when human RBCs are exposed to O_2_ tensions equivalent to 50% hemoglobin oxygen saturation [140]. Further supporting the central role of RBCs in this process, perfusion of blood vessels with RBCs was shown to be necessary to elicit vessel dilation in response to hypoxia [141]. This phenomenon is also supported by exercise data showing that changes in the levels of circulating ATP in blood plasma are proportional to changes in the oxygenation state of hemoglobin in RBCs [137].

A mechanism for ATP release was provided by the detection of functional Panx1 channels in RBCs, and by the lack of Cx43 and vesicular release of ATP in RBCs under physiological conditions (Table 3) [142, 143]. Inhibition of RBC ATP release with the pannexin inhibitors CBX, probenecid, and ^10^Panx later clarified that Panx1 is responsible for ATP release from human RBCs in response to hypoxia, though not for a separate mechanism of ATP release in response to activation of the prostacyclin receptor [144]. Additionally, mechanical deformation of RBCs has been found to induce ATP release via a pathway similar to that of hypoxia-induced ATP release, with impairment of RBC deformability also impairing hypoxic ATP release [140, 145]. The exact mechanism of Panx1 opening in response to hypoxia or RBC deformation remains unsolved, but a variety of data suggests that Panx1 lies downstream of the mechanosensitive G protein-coupled receptor G_i_, (e.g. P2Y receptors), adenylyl cyclase, and cAMP synthesis [140, 144, 146]. However, RBCs are still able to release ATP in response to direct G_i_ stimulation when RBC deformation is prevented. These observations suggest that hypoxia and deformation of RBCs is linked, possibly by G_i_ to initiate the signaling cascade culminating in Panx1-dependent release of ATP into the vessel lumen [145]. The resulting NO that serves as a vasodilator may, in turn, inhibit ATP release in a negative feedback loop [140]. While the mechanism has not been fully explained, this negative feedback is thought to occur via NO-derived inactivation of G_i_ [146]. However, the recent discovery that Panx1 can be inhibited directly by NO via S-nitrosylation on amino acid residues C40 and C346 offers an alternative explanation that must be investigated [90].

**Table 3:** Expression pattern of connexin and pannexin isoforms in red blood cells (RBCs) and platelets. ‘no’ indicates that a protein has yet to be identified in those cells

Protein	RBCs	Platelets	References
Cx32	no	low levels	[147]
Cx37	no	yes	[146, 147]
Cx40	no	yes	[147]
Cx43	no	low levels	[147]
Panx1	yes	yes	[139, 140] / [149, 150]
Panx2	no	no	
Panx3	no	no	

##### Connexins and Pannexins in Platelets

Platelets are anuclear cells found in blood that, in the presence of endothelial damage, bind exposed collagen on the basement membrane of the vessel wall. Further, they are activated to release α-granules that contain a variety of growth and clotting factors and dense granules that contain ADP, ATP, Ca^2+^, serotonin, and histamine; signals that promote further platelet aggregation [147]. Certain pathologic conditions result in elevated platelet activity outside of normal hemostasis, including chronic infectious or inflammatory conditions such as atherosclerosis [147]. When an atherosclerotic plaque ruptures, platelets erroneously become activated, often leading to thrombosis and possible vessel occlusion [148]. Interrupting pathogenic platelet activation and aggregation is therefore an important strategy to prevent and combat thrombosis.

The most common connexin found on the surface of human platelets by immunohistochemistry is Cx37, with lower amounts of Cx32, Cx40, and Cx43 also reported (Table 3) [149, 150]. Platelet α-granule and dense granule secretion was significantly reduced after platelets were stimulated with collagen-related peptide (CRP-XL) in the presence of connexin inhibitors, ^37,43^Gap27, CBX or 18β-GA, suggesting a role for connexin hemichannels during platelet activation [150]. In addition, connexin inhibitors decreased binding of individual platelets to fibrinogen and inhibited platelet aggregation [150]. However, Cx37-deficient mouse platelets did not reveal a significant difference in α-granule and dense granule secretion compared to WT platelets when stimulated with CRP-XL, implicating a connexin other than Cx37 mediates platelet granule secretion [150]. Overall, the findings of Vaiyapuri et al. suggest that connexin hemichannel activity plays an important role in platelet activation and initiation of aggregation via fibrinogen binding.

Interestingly, ^40^Gap27-treated human platelets and Cx40-deficient mouse platelets both showed decreased fibrinogen binding compared to WT when platelets were stimulated with CRP-XL [151]. Inhibition or deletion of Cx40 in platelets also showed significantly decreased expression of P-selectin, a marker of α-granule secretion [151]. These studies may support a role for Cx40 hemichannels, in promoting platelet activation and aggregation. Further investigation of how Cx40 hemichannels may be activated is required.

Panx1 has also been identified on the surface of human platelets, but not Panx2 or Panx3 [152, 153]. Panx1 inhibition or deletion in platelets resulted in impaired collagen-induced aggregation, ATP release, and Ca^2+^ influx [152, 153]. Molica et al. reported that, in platelets, Panx1 co-immunoprecipitates with P2X_1_, an ATP-gated channel involved in thrombosis [152]. Inhibition of P2X_1_ with NF449 decreased collagen-induced platelet aggregation, which was not restored by potassium-stimulated opening of Panx1 channels [152]. Taylor et al. found that the Panx1 inhibitors probenecid and CBX, impaired collagen, thrombin, and TXA_2_ analogue-induced Ca^2+^ influx, possibly through P2X_1_ signaling [153, 154]. Together, these data suggest that P2X_1_ signaling occurs downstream of Panx1 [152–154]. Platelets from patients homozygous for the Panx1-400C polymorphism, which results in a change from glutamine to histamine at residue 5 in the Panx1 amino-terminus, release elevated amounts of ATP at rest when stimulated with K^+^ and demonstrate increased collagen-induced platelet reactivity compared to platelets with the Panx1-400A allele coding for glutamine [152]. In addition, a higher frequency of the Panx1-400C allele was found in cardiovascular patients with hyper-reactive platelets compared to those with hypo-reactive platelets [152]. Further evaluation of connexin- and pannexin-dependent signaling in platelets may provide a new therapeutic target for cardiovascular patients.

## Conclusions

Connexin hemichannels and Panx channels may play an important role in the vascular system, not only in the coordination of endothelial and smooth muscle cell signaling, but also in the control of blood cell function in normal conditions as well as during inflammation. Although several connexin and Panx isoforms are expressed in the vessel wall and blood cells, the signaling mediated by connexin hemichannels and Panx channels in the vascular system seems to rely mainly on channels formed by Cx43 and Panx1, respectively. Both Cx43 and Panx1 have several sites that can be modulated by phosphorylation and S-nitrosylation, which provides mechanisms for rapid and fine regulation of the activity of these channels and for cross talk between the interactions of different signaling pathways.

The most relevant signaling mechanism mediated by connexin hemichannels and Panx channels between the cells of the vascular system is the release of ATP. However, connexin hemichannels and Panx channels are also permeable to Ca^2+^, which is a key second messenger in the control of vasomotor tone by both endothelial cells and smooth muscle cells. In any case, the possible direct contribution of these channels to the regulation of intracellular Ca^2+^ dynamics in the cells of the vessel wall remains to be determined. In addition, although most cells of the vascular system express connexins and Panx channels, it should be noted that the function of these channels is not redundant, as can be observed, for instance, in the vasoconstriction of smooth muscle cells initiated by intravascular blood pressure (i.e. myogenic response) or α1-adrenoceptor activation. The functional specificity of these channels suggests that the manipulation of their activity may provide the opportunity to design novel pharmacological strategies for the treatment of diseases associated with the development of vascular dysfunction or inflammation.

In summary, although multiple connexin and pannexin isoforms have been found in the vasculature as well as the anuclear circulating cells, their channel regulations and functions remain a topic of intense investigation. The separation between connexin hemichannel and pannexin channel function is difficult to ascertain, however it remains vital to explore these mechanisms as there are both convergent and divergent functions throughout the vasculature to maintain homeostasis.
